# Taurine promotes the production of CD4^+^CD25^+^FOXP3^+^ Treg cells through regulating IL-35/STAT1 pathway in a mouse allergic rhinitis model

**DOI:** 10.1186/s13223-021-00562-1

**Published:** 2021-06-19

**Authors:** Jing Zhou, Yi Lu, Wei Wu, Yunhai Feng

**Affiliations:** 1 Department of Otorhinolaryngology, Head and Neck Surgery, Dahua Hospital, Laohumin Road No. 901, Shanghai, 200237 China; 2 Department of Pharmaceutics, School of Pharmacy, Fudan University, Shanghai, 201203 China

**Keywords:** Taurine, CD4^+^CD25^+^FoxP3^+^ T regulatory, IL-35/STAT1, Allergic rhinitis

## Abstract

**Background:**

Allergic rhinitis (AR) is one of the most widespread immune conditions worldwide. However, common treatments often present with significant side effects or are cost-prohibitive for much of the population. A plethora of treatments have been used for the treatment of AR including antihistamines, steroids, and immune modulators. Among the treatments which have shown potential for efficacy in treating AR with a minimum of side effects but remains understudied is the conditionally essential amino acid taurine. Taurine has been previously shown to reduce AR symptoms. Here, we examine the role of taurine in modulating T regulatory cells, modulating the cytokine response in AR, and restoring healthy nasal mucosa.

**Methods:**

Blood samples from 20 healthy donors and 20 AR patients were compared for CD4^+^CD25^+^FoxP3^+^ T regulatory (Treg) cell population percentage, cytokine release, and STAT1 signaling with and without taurine treatment or IL-35 neutralization. An OVA-induced AR mouse model was administered vehicle, taurine, or taurine plus an IL-35 neutralizing antibody and assayed for sneezing frequency, inflammatory cytokine response, nasal mucosa goblet cell density, and T regulatory cell percentage. CD4^+^ cells were further examined for cytokine release, STAT1 phosphorylation, and response to an anti-IL-35 antibody with and without a STAT1 inhibitor.

**Results:**

Comparison of blood from normal donors and AR patients showed a reduction in CD4^+^CD25^+^FoxP3^+^ Treg cells in AR patients and a strong correlation between Treg percentage and IL-35 release. A similar pattern of Treg suppression was found in untreated AR mice when compared to normal control mice wherein there was a reduction in Treg percentage and a corresponding decrease in IL-35 release. AR mice also demonstrated increased sneezing frequency, an infiltration of goblet cell in nasal mucosa, and a reduction in IL-35 release from CD4^+^ cells. Conversely, IL-4, IL-5, and IL-13 secretion from CD4^+^ cells were increased in AR model mice, as was STAT1 phosphorylation. When AR mice were treated with taurine, sneezing frequency and nasal mucosa goblet cell content were reduced while Treg abundance was increased to that of normal mice. Accordingly, IL-35 release was restored, while IL-4, IL-5, and IL-13 secretion from CD4^+^ cells were suppressed. Likewise, STAT1 phosphorylation was inhibited with taurine treatment. Taurine-treated mice also given an IL-35 neutralizing antibody exhibited AR pathology including frequent sneezing and high nasal goblet cell content while retaining a restoration of Tregs. Furthermore, murine AR model CD4^+^ cells exposed to recombinant IL-35 responded with a reduction in inflammatory cytokine release and a decrease in STAT1 phosphorylation, mimicking the effect of taurine treatment.

**Conclusions:**

Taurine induces release of IL-35 in AR; IL-35 promotes the production of CD4^+^CD25^+^FoxP3^+^ Treg cells via a STAT1-dependent pathway. The restoration of Treg populations by taurine normalizes the inflammatory response, reduces AR symptomology, and reduces histopathologic signs of AR.

**Supplementary Information:**

The online version contains supplementary material available at 10.1186/s13223-021-00562-1.

## Background

Allergic rhinitis (AR) is a very common condition with approximately 10–40% people affected globally [1] and is the most widespread chronic disease in children [2], which is manifested by nasal congestion and excessive mucus production, results from a misdirection of the immune system towards non-pathogenic antigens. Frequently, this is airborne pollen with other common allergens being dust or animal dander. While AR is typically thought of a nuisance disorder it can become life-threatening to those with underlying respiratory issues, particularly asthma [3].

At the cellular level AR involves a complex system of immune cell interaction including mast cells [4], T cells [5], macrophages [6], and B cells [7] with an accompanying change in the cytokine and antibody milieu of nasal tissue [8, 9]. The broad range of cellular and molecular actors which caused AR has made the development of treatments difficult as they will need to target multiple pathways which increase the likelihood of side effects.

As AR is a disease of an overactive immune system, treatments are primarily focused on blunting the immune response to reduce symptoms. Common medications include antihistamines [10], corticosteroids [11], anticholerigenics [12], mast cell stabilizers [13], leukotriene inhibitors [14], and anti-IgE immunotherapies [15]. Antihistamines are one of the first therapeutics for the treatment of AR [16] and remain a mainstay of therapy in either oral or topical formulation [1]; however this class of medication is plagued by common side effects such as excessive drowsiness. While corticosteroids are among the most effective treatments for AR, particularly when used in combination with other medications such as antihistamines, they present with rare but potentially serious side effects such as an increase intraocular pressure [17]. Therapeutics which more directly target immune function, such as mast cell and leukotriene inhibitors, typically are more effective with fewer side effects when compared to earlier treatments but can be costly [18]. Regardless, there are no treatments which are universally effective and there remains a clear need for an efficacious AR treatment with a highly tolerable side effect profile.

Taurine, a conditionally essential natural amino acid, has been shown to suppress AR symptoms [19] and is a naturally occurring nutrient with an established safety record [20, 21]. The mechanism by which taurine regulates immune function remains unclear, in part due to the array of related functions found to be regulated by taurine. A central hallmark of autoimmune disease such AR is dysregulation of the immune system resulting in an inappropriate response to benign stimuli. In healthy individuals the immune system is regulated in part by a subset of T cells, T regulatory cells (Treg), which function to inhibit the inflammatory response to prevent a response against the body’s own tissues or harmless foreign bodies. Tregs are primarily identified by the surface markers CD4 and CD25 as well as the transcription factor Forkhead Box P3 (FoxP3) [26–29]. It has been reported that the relative abundance of CD4^+^CD25^+^Foxp3^+^ Tregs was decreased significantly in a Guinea pig model of allergic rhinitis and that increasing the percentage of CD4^+^CD25^+^Foxp3^+^ Tregs restrains allergic reactions through an increase in IL-10 production [30]. In fact, diminution of Treg populations increases the allergic response [31] and AR patients have been shown to have reduced Treg numbers [32–34]. The mechanism of Tregs in the allergic response is is unclear.

Interleukin-35 (IL-35) plays a critical, multifaceted role. IL-35 has been shown to convert resting B and T cells into IL-10 and IL-35 secreting B regulatory (Breg) and T regulatory (Treg) cells which serve to blunt the hypersensitivity type 1 response [35]. Importantly, IL-35 promotes the maturation of naïve T cells via STAT1 and STAT4 signaling to form regulatory iT35 cells which, in turn, secrete additional IL-35 [36].

While taurine has demonstrated effectiveness in AR, and CD4^+^CD25^+^FoxP3^+^ Tregs and IL-35 are known to play a central role in immune response regulation, the association between these components remains to be elucidated.

## Methods

### Human blood samples

A total of 40 human blood samples were used in the present study to evaluate the relationship between IL-35 and CD4^+^CD25^+^FOXP3^+^ Tregs. Blood was obtained from healthy control subjects and from allergic rhinitis patients, n  =  20 for each group. All patients signed written informed consent. This study was approved by the independent ethics committee of Dahua Hospital, Shanghai, China and strictly obeyed the Declaration of Helsinki.

### Mouse model

Eight-week-old C57BL/6 mice (n  =  48, n  =  12/treatment group), free of murine-specific pathogens, were obtained from the animal department at Shengjing Hospital, China Medical University (Shenyang, China). The mice were housed in a controlled environment with a 12/12-h light/dark cycle with free access to food and water. They were maintained on an ovalbumin (OVA)-free diet. The experimental procedures were approved by the ethical committee of Dahua Hospital, Shanghai, China. All mice were handled according to Institutional Animal Care and Use Committee (IACUC) guidelines and experiments were conducted following the institute’s guidelines for animal experiments.

The allergic rhinitis model mice were sensitized by intraperitoneal (i.p.) injection with 1 mg/mL OVA (Sigma-Aldrich, St. Louis, MO, USA) and 20 mg/mL aluminum hydroxide (Sigma-Aldrich) in normal saline at a dose of 100 μL/mouse. The control group mice were sensitized and challenged with saline. Taurine (3% w/v) and IL-35 antibody (10 ng/ml) were used to treat mice through tail intravenous injection, respectively. Then, then the sneezes were counted. All mice were sacrificed via cervical dislocation at day 42 after injection, and nasal mucosa tissues were removed from the xenograft mice and fixed in 4% formalin for further analysis.

### Human or mouse CD4^+^ T cell isolation

Human or mouse blood samples were diluted by PBS solution (1:1) and peripheral blood mononuclear cells were obtained by centrifugation on a lymphocyte separation medium. The concentration of lymphocytes was then adjusted to 1 × 10^6^/mL. Human or mouse CD4^+^ T cells were isolated from PBMC using CD4^+^ T cell isolation kits respectively [130-096-533 (human) and 130-104-454 (mouse), Miltenyi Biotec, Germany]. Manufacturer instructions were followed for all procedures.

### Cell culture

Human PMBC cells were grown in DMEM (Trueline, Kaukauna, WI, USA) supplemented with 10% FBS (Thermo Fisher Scientific, Waltham, MA, USA) 2 mM L-glutamine (Solarbio, Beijing, Peoples’ Republic of China), and 1% penicillin/streptomycin (Solarbio, Beijing, Peoples’ Republic of China) and were maintained under a 5% CO_2_ atmosphere, at 37 °C, and then used to isolate CD4^+^ T cells The connection between taurine and IL-35 was examined by using taurine (20 mmol/L) and IL-35 recombinant protein (10 ng/mL) d, then supernatants were collected and stored in − 20 for further use in ELISA. The STAT1 inhibitor fludarabine phosphate (Fludara, 50 μmol/L, A8317, APEXBIO, USA) was dissolved in DMSO (D2650, Sigma, USA) was used block the endogenous expression of STAT1 in cells.

### ELISA

IL-4, IL-5, IL-13, and IL-35 quantitative ELISA kits were obtained from Bioscience (Shjgogo, Shanghai, China) and used to determine the concentration of IL-4, IL-5, IL-13, and IL-35 released from mouse CD4^+^ T cells. All procedures were performed according to the protocol of the manufacturer. Briefly, biotin-labeled antibodies against IL-4, IL-5, IL-13, or IL-35 were incubated with cell supernatant in a 96-well ELISA plate at 37 °C for 2 h. The plate was washed five times with wash buffer and incubated with HPR-labeled avidin and re-washed. Detection was provided by colorimetric reaction (≤  5 min) which was read on a microplate reader (Pulangxin, China) to determine the OD450 value. All samples were analyzed in triplicate.

### Western blot

Whole protein lysates were extracted from indicated cells by RIPA lysis buffer (JRDUN, Shanghai, China) with EDTA-free protease inhibitor cocktail (Roche, Mannheim, Germany). Protein concentration was quantified by an enhanced BCA protein assay kit (Thermo Fisher, Waltham, MA, USA). Equal mass of total protein (25 μg) were fractionated on 10% SDS-PAGE and transferred to a nitrocellulose membrane (Millipore, Temecula, CA, USA) overnight. After being blocked with 5% nonfat dry milk for 1 h at room temperature, the membranes were probed at 4 °C overnight with primary antibodies followed by secondary antibody anti-mouse IgG (1:1000; Beyotime, Shangai, China) for 1 h at 37 °C. Enhanced chemiluminescence system (Tanon, Shanghai, China) was used to detect protein abundance. The primary antibodies used are as follows: IL-35 (1:500, 701101, Invitrogen, Carlsbad, CA, USA), Foxp3 (1:500, ab75763, Abcam, Cambridge, UK), STAT1 (1:1000, ab31369, Abcam, Cambridge, UK), p-STAT1 (1:1000, ab4742, Abcam, Cambridge, UK), GAPDH (1:1000, CST, Danvers, MA USA).

### Flow cytometry (FCM) assay

Human or mouse mononuclear cells were grown in RMPI-1640 medium containing 10% fetal bovine serum (FBS; Gibco, New York, USA), penicillin (100 μL/mL; Solarbio, Beijing, China) and streptomycin (100 g/mL; Solarbio, Beijing, China) and used for FCM assay.The Anti-CD4 FITC [11-0049-42 (human) and 11-0043-82 (mouse), Ebioscience, San Diego, CA, USA)], Anti-CD25 APC [17–0259-42 (human)17–0251-82 (mouse), Ebioscience, San Diego, CA, USA] and Anti- Foxp3 PE [12–4776-42 (human) and 12–5773-82 (mouse), Ebioscience, San Diego, CA, USA] were separately added (5 µL) to the diluted lymphocytes according to the manufacturer’s instructions (Ebioscience, San Diego, CA, USA). A FACS Calibur flow cytometer (Becton Dickinson, San Jose, CA, USA) was used to establish FCM and data analysis was performed using CellQuest software (Becton Dickinson, Bedford, MA, USA). Cells treated with a non-specific secondary antibody only were defined as blank control for 48 h. The scatterplots are arranged as follows: the upper left quadrant contains CD4^+^CD25^−^FOXP3^+^ cells, the lower left quadrant contains CD4^+^CD25^−^FOXP3^−^ cells, while the upper and lower right contain CD4^+^CD25^+^FOXP3^+^ and CD4^+^CD25^+^FOXP3^−^ cells, respectively.

### Histopathology assay

All sample tissues were fixed in 10% formalin for 48 h and subsequently embedded in paraffin blocks and cut into slices using a microtome (Leike, Wuhan, China). Slides were deparaffinized and rehydrated in a xylene bath followed by ethanol mixed with increasing concentrations of water. Then, slices were exposed to hematoxylin and eosin (H&E) staining. Three random fields on each slide were observed.

### Statistical analysis

GraphPad Prism software Version 7.0 (La Jolla, CA, USA) was used for statistical analyses. Data were displayed as mean  ±  SD for a minimum of three replicates. Statistical significance was determined by one-way ANOVA for multiple comparisons. A P value  <  0.05 indicates statistical significance.

## Results

### IL-35 secretion correlates positively with the percentage of CD4^+^CD25^+^Foxp3^+^ Treg cells in the blood of patients with allergic rhinitis (AR)

**Fig. 1:**
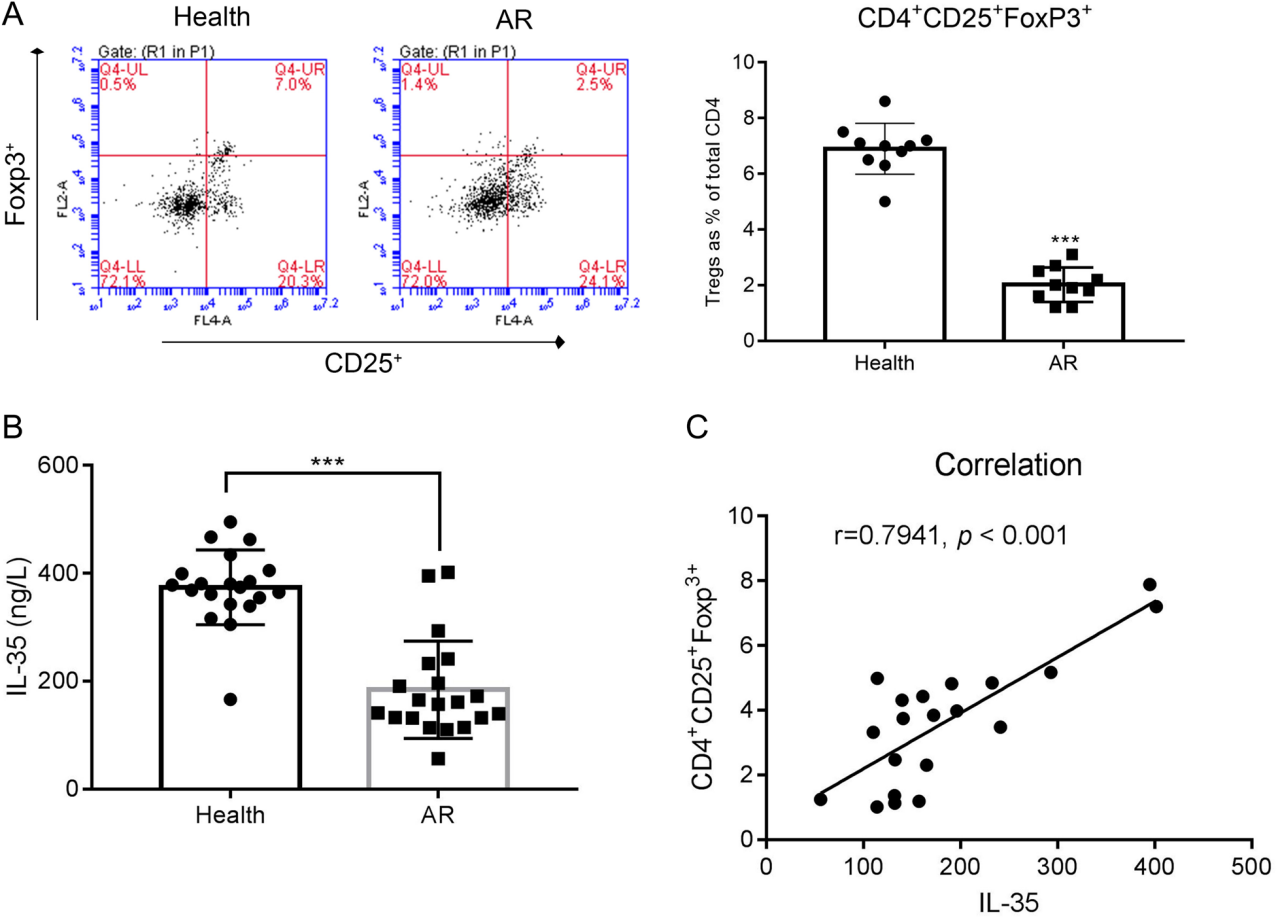
IL-35 was positively correlated with the percentage of CD4  +  CD25  +  Foxp3  +  Treg cells in the blood of patients with allergic rhinitis (AR). **A** The percentage of CD4  +  CD25  +  Foxp3  +  Treg cells was downregulated in AR patients compared with that in health samples. ***p  <  0.001 vs Health. **B** The secretion of IL-35 was reduced in AR patients compared with health. ***p  <  0.001 vs Health. **C** Correlation analysis between IL-35 and the percentage of CD4  +  CD25  +  Foxp3  +  Treg cells

PBMCs were isolated from whole blood of patients with AR or from healthy controls and magnetically sorted for CD4 expression followed FoxP3 and CD25 quantification by flow cytometry to identify Tregs. CD25^+^/FoxP3^+^ cells were significantly less abundant in AR patients when compared to normal controls (2.5 vs 7%); interestingly the reduction in double-positive cells can be accounted for by the loss of FoxP3 expression alone (Fig. 1A). Moreover, IL-35 secretion from cultured PBMCs isolated from AR patients was significantly reduced compared to that from that secreted by PBMCs isolated from healthy controls, as measured by ELISA (Fig. 1B, left). We found a strong positive correlation (r  =  0.7941, p  <  0.001) between the percentage CD4^+^CD25^+^FoxP3^+^ cells and IL-35 secretion (Fig. 1B, right).

### Antibody targeting IL-35 partly disrupted the function of taurine in a mouse AR model

**Fig. 2:**
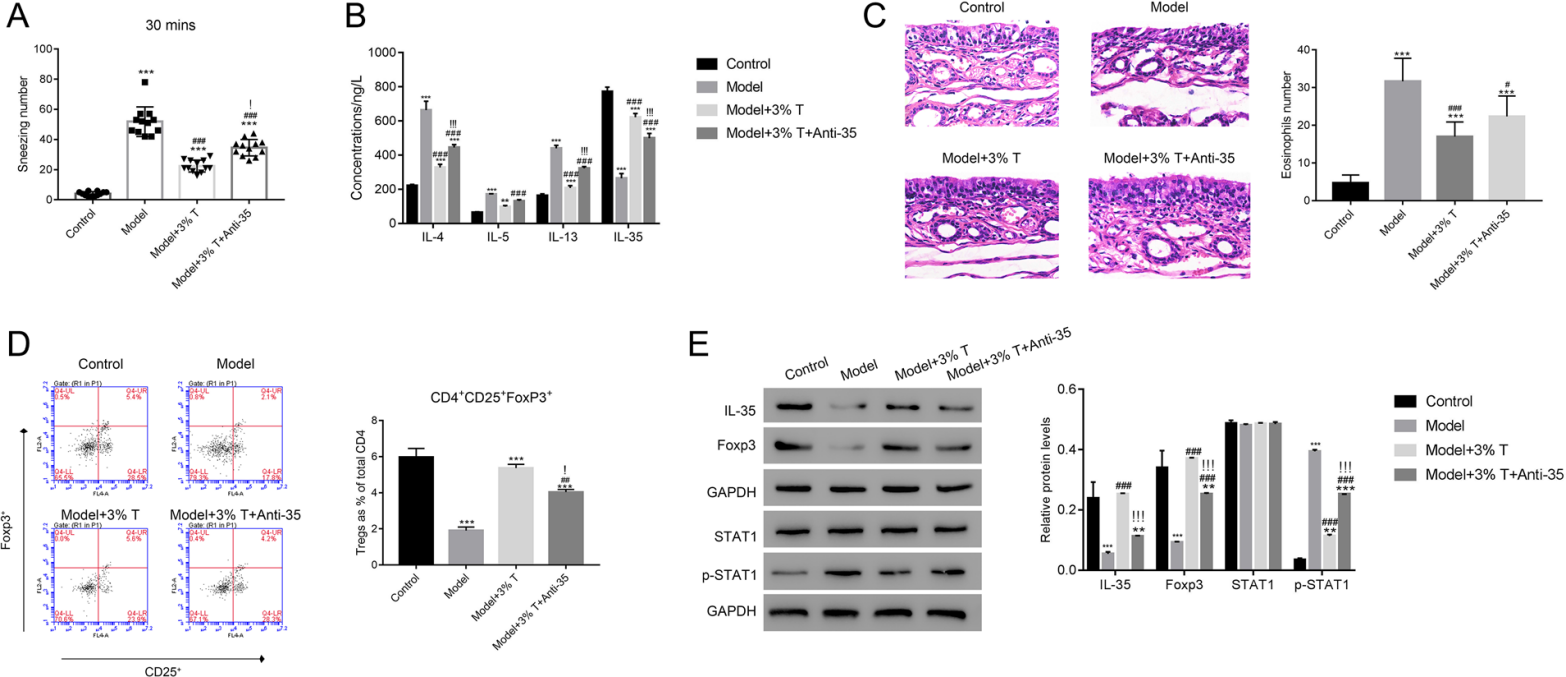
IL-35 antibody partly disrupted the function of taurine in mouse AR model. **A** Taurine treatment reduced the sneezing number of model mouse. ***p  <  0.001 vs Control; ###p  <  0.001 vs Model; !p  <  0.05 vs Model  +  3% T. T: Taurine. **B** The concentrations of IL-4, IL-5, IL-13 and IL-35 were examined in different group as indicated. **p  <  0.01 vs Control, ***p  <  0.001 vs Control; ###p  <  0.001 vs Model; !p  <  0.05 vs Model  +  3% T. **C** Taurine treatment alleviated the AR-induced injury in mouse. **D** The percentage of CD4  +  CD25  +  Foxp3  +  Treg cells was increased in model group with Taurine treatment. ***p  <  0.001 vs Control; ##p  <  0.01 vs Model; !p  <  0.05 vs Model  +  3% T. **E** Western blot was used to examine the protein contents of IL-35, Foxp3, STAT1 and p-STAT1 in different nasal mucosal tissues as indicated. **p  <  0.01 vs Control, ***p  <  0.001 vs Control; ###p  <  0.001 vs Model; !!!p  <  0.001 vs Model  +  3% T

To investigate the interplay between taurine and the immunoregulatory cascade, we administered taurine with and without co-administration of an IL-35 neutralizing antibody to an OVA-induced allergic rhinitis mouse model. Mice was sensitized with only saline were used as a non-AR control and saline-treated AR mice served as a vehicle-treated control. First, sternutation was quantified for a 30-min observation period as marker for general AR symptomology. Saline-sensitized negative control mice exhibited minimal sneezing. As expected, saline (vehicle) treated AR mice demonstrated frequent sneezing, a significant increase over control mice (*p * <  0.001). AR mice treated with taurine, however experienced a robust inhibition (*p * <  0.001). To establish a potential role of IL-35 in this AR model an IL-35 neutralizing antibody was administered to AR mice also treated with taurine; IL-35 neutralization restored AR symptoms to nearly that of vehicle treated AR mice (*p * <  0.05) (Fig. 2A) strongly implicating IL-35 as mediator of sneezing in AR mice treated with taurine.

To delve in the mechanistic impact of cytokine release in a taurine-treated AR model, the concentrations of IL-4, IL-5, IL-13, and IL-35 were measured. As expected, the serum concentration of IL-4, IL-5, and IL-13 were increased in the AR model compared to the saline control mouse group indicating an immune response (Fig. 2B; p  <  0.001). In alignment with a reduction in AR symptoms, the concentrations of these cytokines decreased following taurine treatment (Fig. 2B; p  <  0.05) Notably, the concentration of immunoregulatory IL-35 decreased significantly in the AR model and was restored to a nearly normal concentration with taurine treatment.

To verify the role of IL-35 in attenuating AR following taurine treatment, an anti-IL-35 antibody was employed to sequester the cytokine in mice also given taurine. Following anti-35 antibody treatment IL-4, IL-5, and IL-13 concentrations were partially restored (*p * <  0.05), indicating the anti-inflammatory effects of taurine were lost and suggesting taurine suppresses AR via an IL-35-dependent mechanism (Fig. 2B). Moreover, the resulting serum concentration of IL-35 in taurine plus antibody treated AR mice was lower compared to the taurine alone condition (Fig. 2B).

### Taurine alleviates AR damage to nasal mucosa

Comparison of H&E stains of nasal mucosa from vehicle treated AR mice show extensive infiltration of mucus-secreting goblet cells indicating both a successful creation of an AR model and providing a tissue-level explanation for the increase in sneezing noted above. In contrast, taurine-treated AR mice were found to have mucosa histology which was highly similar to that of non-AR (healthy) control mice with minimal goblet cell presence. Administration of an anti-IL-35 antibody in combination with taurine treatment in AR mice resulted in goblet cell-rich mucosa with morphology nearly identical to that of the saline-treated AR mice (Fig. 2C). Moreover, the number of Eosinophils was also significantly downregulated with IL-35 antibody treatment in the presence of taurine. In addition, we sought to examine the effects of taurine and anti-IL-35 antibody treatment on the Treg population.

As shown in Fig. 2D, the percentage of CD4^+^CD25^+^FoxP3^+^ Tregs within the CD4^+^ population decreased from approximately 6% in non-AR mice to 2% in AR model mice. Administration of taurine rescued the Treg population in AR mice to nearly 6% of CD4^+^ cells. Moreover, IL-35 antibody plus taurine treatment inhibited the increase in Treg numbers induced by taurine, resulting in approximately 4% CD4^+^CD25^+^FoxP3^+^ indicating taurine restores Treg populations though an induction of IL-35 (Fig. 2D).

As expected, both IL-35 and FoxP3 protein levels were reduced in the AR model compared to control mice, while STAT1 phosphorylation was increased. Taurine treatment rescued IL-35 and FoxP3 levels and reduced STAT1 phosphorylation to nearly that of non-AR tissue (Fig. 2E). To delve into the role of IL-35 on protein abundance we examined FoxP3 and pSTAT1 in nasal mucosa tissue from mice treated with both taurine and an anti-IL-35 antibody. FoxP3 levels were reduced compared to taurine-treated AR mice while STAT1 phosphorylation was increased indication a partial reinstatement of the AR model state (Fig. 2E).

### IL-35 targeting antibody disrupted taurine response in AR CD4  +  T cells

**Fig. 3:**
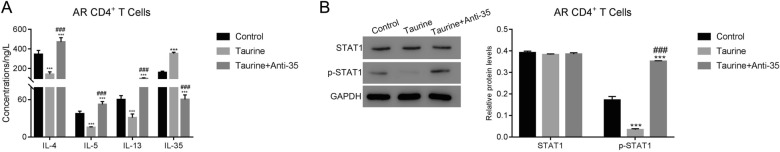
IL-35 antibody disrupted the function of Taurine in AR CD4  +  T cells. **A** The concentrations of IL-4, IL-5, IL-13 in taurine treated AR CD4  +  T cells were increased in the presence of IL-35 antibody. ***p  <  0.001 vs Control; ###p  <  0.001 vs Taurine. **B** IL-35 antibody promoted the phosphorylation of STAT1 in taurine treated AR CD4  +  T cells. ***p  <  0.001 vs Control; ###p  <  0.001 vs Taurine

Next, taurine was used to culture human PBMCS. As shown in Additional file 1: Figure S1A, the secretion of IL-35 was significantly upregulated in human PBMC in the presence of taurine. Moreover, taurine treatment also deeply inhibited the phosphorylation of STAT1 (Additional file 1: Figure S1B).We sought to delineate the interplay of taurine and IL-35 on CD4^+^ AR cells. Murine AR CD4^+^  cells were exposed to taurine with or without an anti-IL-35 antibody. Secretion of the pro-inflammatory cytokines IL-4, IL-5, and IL-13 were all reduced with taurine treatment suggesting a reduction in AR pathology. In contrast, anti-inflammatory IL-35 secretion was increased. Each of these effects were reversed when an IL-35 neutralizing antibody was administered with the taurine treatment, a clear indication that the anti-inflammatory impact of taurine is mediated via IL-35 (Fig. 3A). Accordingly, STAT1 phosphorylation was strongly reduced in CD4^+^  cells following taurine treatment and was fully restored when cells were cultured in the presence of an IL-35 neutralizing antibody (Fig. 3B).

### Recombinant IL-35 mimics taurine in AR CD4^+^ T cells

**Fig. 4:**
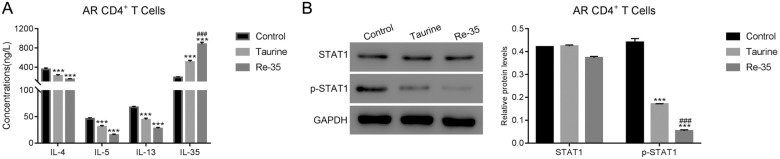
The recombinant protein of IL-35 presented the similar function as Taurine in AR CD4  +  T cells. **A** The concentrations of IL-4, IL-5 and IL-13 were deeply suppressed in Taurine or IL-35 recombinant protein (Re-35) cultured AR CD4  +  T cells. ***p  <  0.001 vs Control; ###p  <  0.001 vs Taurine. **B** Taurine or Re-35 deeply suppressed the phosphorylation of STAT1 in AR CD4  +  T cells. ***p  <  0.001 vs Control; ###p  <  0.001 vs Taurine

To elucidate the role of IL-35 in regulating the autoimmune cytokine cascade, recombinant IL-35 or taurine was administered to CD4^+^ cells isolated from AR mouse model blood. Measurement of the type 2 cytokines IL-4, IL-5, and IL-13 showed all were reduced significantly with either taurine or recombinant IL-35 treatment (*p * <  0.001; Fig. 4A). Recombinant IL-35 also induced further IL-35 secretion (Fig. 4A). Moreover, both taurine and recombinant IL-35 reduced STAT1 phosphorylation (Fig. 4B).

### STAT1 inhibition suppressed the IL-35 antibody function in normal CD4 T cells

**Fig. 5:**
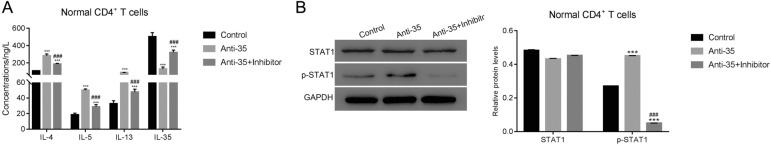
The STAT1 inhibitor suppressed the function of IL-35 antibody in normal CD4  +  T cells. **A** The concentrations of IL-4, IL-5 and IL-13 in IL-35 antibody cultured normal CD4  +  T cells were downregulated with the treatment of STAT1 inhibitor. ***p  <  0.001 vs Control; ###p  <  0.001 vs Anti-35. **B** The STAT1 inhibitor significantly suppressed the phosphorylation of STAT1 in anti-35 cultured normal CD4  +  T cells. ***p  <  0.001 vs Control; ###p  <  0.001 vs Anti-35

To further investigate the role of STAT1 in immunoregulation, we employed a STAT1 inhibitor, fludarabine phosphate, in conjugation with anti- IL-35 antibody treatment of normal mouse CD4^+^ cells. As expected, exposure to anti-IL-35 antibody alone produced an increase in secretion of IL-4, IL-5, and IL-13 (*p * <  0.001; Fig. 5A). Treatment with the STAT1 inhibitor along an anti-IL-35 antibody diminished the response to the antibody and resulted in a reduction in cytokine release (*p * <  0.001; Fig. 5A). Accordingly, western blotting showed an increase in STAT1 phosphorylation (activation) in cells subjected to antibody treatment which was reversible with STAT1 inhibitor treatment (Fig. 5B).

## Discussion

In this study we described the interplay between taurine and IL-35 in moderating AR symptoms. While taurine has been known to relieve AR symptoms [19, 22], we were able to describe this mechanism at multiple physiological levels. AR mice treated with taurine experience reduced sneezing frequency owing to a reduction in goblet cell infiltration into the nasal mucosa and taurine induced an expansion of IL-35 secreting CD4^+^CD25^+^FoxP3^+^ Tregs. IL-35 reduced STAT1 phosphorylation and decreases inflammatory cytokine release from CD4^+^ cells.

Taurine is a known immunoregulatory agent which has been shown to be impactful in several disorders [42–44] in addition to AR [19, 22, 45] however the bulk of this work has been broad and lacked an examination of detailed cellular mechanisms. The research presented here is, to the best of our knowledge, the first to focus on a detailed mechanistic analysis of taurine in AR. We believe that it is through this detailed approach will be promote the development of novel treatments based on the pathway used by taurine.

Secreted by CD4^+^FoxP3^+^ Treg cells, IL-35 plays a central role in AR. IL-35 has a well-defined inhibitory function on AR symptomology [46], indeed serum IL-35 levels are inversely correlated with the total nasal symptom score (TNSS) in children with AR [47], and recombinant IL-35 has been used successfully as an exploratory therapeutic in an OVA mouse AR model [48]. Our results align well with these data in that serum and tissue IL-35 levels are inversely correlated to sneezing, goblet cell infiltration of nasal mucosa, and inflammatory cytokine release resulting from AR. Mechanistically, we found that IL-35 had an inhibitory effect on STAT1 phosphorylation in AR cells, in agreement with previous research showing a role for STAT1 in murine AR, in particular for the regulation of cytokine secretion [49] and with other research showing a decrease pSTAT1 abundance following coadministration of IL-35 and TNF-α  +  IL-2β [41].

Our results can be used to form a framework for the development of AR therapeutics; knowing that the critical downstream mediators of taurine-mediated AR suppression are CD4^+^CD25^+^FoxP^+^ Treg cells, IL-35, and STAT1 can facilitate the development of agents such as drug-like taurine analogs or other compounds which target this pathway. Other such modalities can include immunotherapies which target IL-35 or its receptor in AR. With the increasing use of recombinant antibodies as therapeutics, both in the autoimmune and oncology spaces, we indeed envision a role for IL-35 in the arsenal of AR medications.

As the human immune system is nearly infinitely complex, there are factors which may play a role in the taurine/IL-35/Treg pathway which may remain important but are outside cell interactions of the scope of this work. These include cAMP, which is enriched in Tregs and functions through direct cell: to mediate immune regulation [50], CTLA-4, a well-described immune checkpoint protein [51], and the immuno-oncology target PD-1 [52]. Moreover, we found that anti-IL-35 treatment only partially reversed the effects of taurine, suggesting an additional immunoregulatory pathway may be involved in AR suppression. We verified the importance of FoxP3^+^ Treg cells in AR here, future research will be required to determine if there is any consequential difference between FoxP3^hi^ vs FoxP3^Lo^ Treg cells [53].

In the present study, taurine was administrated to mice through tail intravenously injected and found that taurine was a promising agent in the treatment for AR. However, there was no evidence to illustrate that the taurine can be administrated to human in the same way as indicated. Therefore, the following analyses can be focused to explore the way for taurine administration in human. Moreover, a larger clinic population samples will be necessary to prove our findings in the present study.

## Supplementary Information

**Additional file 1: Figure S1.** Taurine treatment promoted the secretion of IL-35 and inhibited the phosphorylation of STAT1 in human PBMCS. A. ELISA was used to examine the secretion of IL-35 in human PBMCS that co-cultured with taurine. ***p < 0.001 vs vehicle. B. Western blot was used to examine the relative protein levels of STAT1 and p-STAT1. ***p < 0.001 vs vehicle.

## Data Availability

The datasets used and/or analysed during the current study are available from the corresponding author on reasonable request.

## Abbreviations

AR: Allergic rhinitis
Treg: T regulatory
FoxP3: Forkhead Box P3
Breg: B regulatory
OVA: Ovalbumin
FCM: Flow cytometry
